# The development of a Consensus Conference on Pediatric Procedural Sedation in the Emergency Department in Italy: from here where to?

**DOI:** 10.1186/s13052-020-0812-x

**Published:** 2020-05-01

**Authors:** Idanna Sforzi, Silvia Bressan, Claudia Saffirio, Salvatore De Masi, Leonardo Bussolin, Liviana Da Dalt, Fabio De Iaco, Itai Shavit, Baruch Krauss, Egidio Barbi, on behalf of the Procedural Sedation Analgesia Consensus working group, Ilaria Bergese, Klaus Peter Biermann, Fabio Borrometi, Lorenzo Calligaris, Barbara Cantoni, Silvia Fontanazza, Diego Fornasari, Chiara Ghizzi, Mirco Gregorini, Mario Guarino, Manuela L’Erario, Giovanna La Fauci, Alberto Lai, Simone Lazzeri, Maria Carmela Leo, Ersilia Lucenteforte, Ada Macchiarini, Stefano Maiandi, Massimo Mandò, Alessandro Mazza, Giovanni Montobbio, Alessandro Mugelli, Roberta Parrino, Marina Sammartino, Jürgen Schleef, Angelica Spotti, Caterina Tomasello, Monica Toraldo di Francia, Chiara Trapani, Marcella Turini, Laura Vagnoli, Simona Vergna, Gianni Virgili, Giovanni Vitali Rosati, Davide Zanon

**Affiliations:** 1 Pediatric Emergency Department and Trauma Center, Meyer Children’s Hospital, Viale Pieraccini 24, 50139 Florence, Italy; 2 Department of Women’s and Children’s Health, University of Padova, Padova, Italy; 3 Health Sciences Department, University of Florence, Meyer University Children’s Hospital, Florence, Italy; 4 Emergency Department, Martini Hospital, ASL Città di Torino, Torino, Italy; 5 Pediatric Emergency Department, Rambam Health Care Campus, Haifa, Israel; 6 Division of Emergency Medicine, Boston Children’s Hospital, and the Department of Pediatrics, Harvard Medical School, Boston, MA USA; 7 Institute for Maternal and Child Health IRCCS “Burlo Garofolo”, Trieste, Italy; 8 University of Trieste, Trieste, Italy

**Keywords:** Pediatric, Procedural sedation and analgesia, Consensus, Emergency department

## Abstract

**Background:**

In Italy, as in many European countries, Pediatric Emergency Medicine is not formally recognized as a pediatric subspecialty, hindering nation-wide adoption of standards of care, especially in the field of procedural sedation and analgesia (PSA) in the Emergency Department (ED). For this reason PSA in Italy is mostly neglected or performed very heterogeneously and by different providers, with no reference standard. We aimed to describe the procedures and results of the first multidisciplinary and multi-professional Consensus Conference in Italy on safe and effective pediatric PSA in Italian EDs.

**Methods:**

The preparation, organization and conduct of the Consensus Conference, held in Florence in 2017, followed the recommended National methodological standards. Professionals from different specialties across the country were invited to participate.

**Results:**

Overall 86 recommendations covering 8 themes (pre-sedation evaluation, pharmacologic agents, monitoring, equipment and discharge checklists, training, non-pharmacologic techniques, the adult ED setting, impact on hospitalizations) were developed, taking into account the Italian training system and healthcare organization characteristics.

**Conclusion:**

The results of the first multidisciplinary and multi-professional Consensus Conference in Italy are meant to provide up-to-date national guidance to improve the standard of care of children undergoing painful and stressful procedures in the ED. The recommendations will be periodically updated as new relevant evidence is published.

## Introduction

Children presenting to the emergency department (ED) often need painful, uncomfortable or stressful procedures or painless imaging that require immobility as part of their diagnostic workup or treatment [1–5]. Cooperation of children may be variable and is related to the level of painful stimulus, as well as to their developmental and anxiety level [6, 7]. Relief of procedural pain and anxiety in children is an ethical imperative given the short and long-term physical, physiological and psychological effects if left untreated [8]. To ensure successful completion of diagnostic and therapeutic procedures, while avoiding distress, procedural sedation and analgesia (PSA) is often required in the ED [9]. As such, PSA has long been standard practice to facilitate procedures for children in the ED in many countries [4, 10–16].

Ability to provide PSA in the ED allows for faster completion of procedures, less distressful transitions of the patients between different hospital teams, shorter hospital stay, a better use of resources and overall cost savings [17, 18].

Expertise in PSA is a core competency in Emergency Medicine (EM) and Pediatric Emergency Medicine (PEM) training programs in countries where these specialties are formally recognized, such as in the United States, Canada, Australia, the United Kingdom [19–24] and more recently in other European countries (e.g. Switzerland) [20]. EM and PEM-trained physicians have specific skill sets to manage the airways and ventilation that are necessary to provide patient rescue and are fully qualified to administer/provide all levels of analgesia/sedation [4, 5, 9, 16, 25]. Traditionally, pediatric PSA has been provided in many centers by anesthesiologists due to their specialist skills. However, the operating room may not be easily accessible in a timely fashion from the ED, Anesthesia cover is variable from center to center, their pediatric skill set may also be variable and their involvement to provide PSA for ED patients may not always be an appropriate use of resources considering the competing tasks they are allocated to in the hospital [26–28].

In Italy, EM has been formally recognized as a specialty since 2009 [29], when the first residency program started. EDs had been staffed by different professionals (e.g. internal medicine specialists, surgeons, anesthesiologists) until EM trained physicians first graduated. PEM is not yet a formally recognized sub-specialty in Italy. Despite this, PEM is practiced in some tertiary care pediatric centers in the country, with some variability in ED organization models.

With respect to training, PSA is not formally included in the Italian EM curriculum. In addition, despite the fact that a substantial proportion of children are seen in community EDs staffed by emergency physicians [30], their training in pediatrics is very limited. As for PEM, training in the pediatric ED is a mandatory requirement to be certified as a specialist in Pediatrics. However, there are only general principles guiding the training of pediatricians interested in becoming PEM physicians and no specific recommendations on training requirements exist to acquire the necessary PSA skill set [31]. This lack of national standards [32] and curriculum leads to heterogeneous, sub-optimal non-standard provision of PSA for children in Italian EDs [33].

To fill this gap in Italy, we set out to develop a national consensus on PSA in the ED setting, with the aim of improving the standard of care of children undergoing procedures in the ED and to support the development of hospital policies based on national documents.

## Material and methods

In June 2016, the idea of holding a consensus conference (CC) on ED-PSA in children was conceived and subsequently planned, according to the recommended national methodological standards issued by the Italian Ministry of Health [34].

The CC organizers (I.S. and representatives of the Meyer Children’s Hospital) nominated the Technical Scientific Committee (TSC). The TSC included methodology and literature search experts from the Meyer Children’s Hospital’s Clinical Trial Office (Including S.DM), as well as six pediatric emergency physicians, from different Italian pediatric centers, with expertise in pediatric sedation and analgesia in the emergency department and training in the synthesis and appraisal of scientific papers (including I.S., S.B., C.S.) for the review of relevant articles.

The CC organizers, together with the Technical Scientific Committee (TSC), drew up clinical questions that covered eight main themes, based on both the most recent NICE guidelines “Sedation in children and young people” [4, 5] (five questions) and on specific needs related to the Italian setting (three questions) (Table 1).

**Table 1:** Clinical themes and questions

**Q1** P for children and young people under the age of 18 undergoing diagnostic and therapeutic procedures under procedural sedation and analgesia (PSA) in the Emergency Department (ED) provided by non-anesthetists I what are the factors that determine C / O eligibility to receive PSA? and what is the role of fasting with respect to eligibility for PSA in the ED? • Which factors should be assessed to justify the use of PSA, rather than no sedation or general anesthesia? • What validated tools should be used to support assessment? • Who should make the assessment and how should the assessment be recorded? • How should the consent for PSA be obtained? • Should fasting versus no fasting be implemented to prevent adverse outcomes?	
**Q2** P for children and young people under the age of 18 undergoing diagnostic and therapeutic procedures under PSA in the ED provided by non-anesthetists I is the administration of midazolam/opioids/nitrous oxide/ketamine/propofol/dexmedetomidine C compared with usual care/analgesia alone/another sedation drug/psychological technique/general anesthesia O safe and effective? • Is midazolam (with or without: analgesia, another drug or psychological techniques) effective and safe for sedation (at minimal, moderate, and deep levels) in comparison with usual care, with analgesia alone, with another sedation drug, with psychological techniques or with general anesthesia? • Are opioids (with or without: analgesia, another drug or psychological techniques) effective and safe for sedation (at minimal, moderate, and deep levels) in comparison with usual care, with analgesia alone, with another sedation drug, with psychological techniques or with general anesthesia? • Is 50% nitrous oxide premixed with 50% O_2_ (with or without: analgesia, another drug or psychological techniques) effective and safe for sedation (at minimal, moderate, and deep levels) in comparison with usual care, with analgesia alone, with another sedation drug, with psychological techniques or with general anesthesia? • Is ketamine (with or without: analgesia, another drug or psychological techniques) effective and safe for sedation (at minimal, moderate, and deep levels) in comparison with usual care, with analgesia alone, with another sedation drug, with psychological techniques or with general anesthesia? • Is propofol (with or without: analgesia, another drug or psychological techniques) effective and safe for sedation (at minimal, moderate, and deep levels) in comparison with usual care, with analgesia alone, with another sedation drug, with psychological techniques or with general anesthesia? • Is dexmedetomidine (with or without: analgesia, another drug or psychological techniques) effective and safe for sedation (at minimal, moderate, and deep levels) in comparison with usual care, with analgesia alone, with another sedation drug, with psychological techniques or with general anesthesia?	
**Q3** P for children and young people under the age of 18 undergoing diagnostic and therapeutic procedures under PSA in the ED provided by non-anesthetists I what are the systems and timing of the monitoring and assessment tools for PSA C / O more appropriate/useful for each type of PSA?	
**Q4** P for children and young people under the age of 18 undergoing diagnostic and therapeutic procedures under PSA in the ED provided by non-anesthetists I what are the available/validated checklists C / O for safe conduct of PSA and safe discharge?	
**Q5** P for non-anesthetists providers of PSA in the ED for children and young people under the age of 18 undergoing diagnostic and therapeutic procedures I what are the necessary training requirements at an institutional and national level C / O to be able to perform safe and effective PSA? • What generic and specific skills are required for different team members and for different levels of sedation? What training and competences are required? • Who should train the nurses, doctors and pediatricians of the ED?	
**Q6** P for children and young people under the age of 18 undergoing diagnostic and therapeutic procedures under PSA in the ED provided by non-anesthetists I what are the effective strategies C / O for successful implementation of non-pharmacologic techniques? • What standard psychological preparation, coping skills and strategies should be used? • Can a combination of psychological techniques and sedative drugs help reduce the doses of sedatives? • What instruments can be used to implement the use of the non-pharmacologic techniques?	
**Q7** P for adult Emergency Medicine doctors providing PSA in the ED for children and young people under the age of 18 undergoing diagnostic and therapeutic procedures I what (if any) differences in practice should be applied C / O for safe and effective PSA? • In which way should PSA provided by adult ED physicians, from pre-assessment to discharge, be distinguished from PSA administered by pediatricians/pediatric emergency physicians and how should the differences be managed?	
**Q8** P for children and young people under the age of 18 undergoing diagnostic and therapeutic procedures in the ED I is administration of PSA in the ED C compared to no PSA administration O effective in optimizing healthcare costs/resource use at a local and a national level and in improving patient experience? • What impact could the performance of diagnostic and therapeutic procedures under PSA in the ED have on costs (for the patient, for the Institution, for the National Health System)?	

Questions on themes 1 to 5 are adapted from the NICE guidelines [4]

According to the reference methodological standards, the CC organizers and the TSC also selected the “Expert Panel” (EP), including expert professionals with the role of presenters of the evidence for each clinical question, and discussants to favor the discussion and debate at the CC. The EP was composed of professionals with a recognized expertise in the field (hinged on their established expertise as sedation researchers, educators and clinical leaders) coming from all over the Country to reflect its practice and geographic diversity. To ensure a multidisciplinary and multi-professional representativeness of the EP, professionals were invited from the fields of Adult and Pediatric Anesthesiology, Intensive Care, Emergency Medicine, Pediatric Emergency Medicine, Pediatrics, Nursing, and Psychology.

In preparation of the CC, the TSC also conducted a systematic literature search with the support of an expert librarian to retrieve relevant references published after the end date of the literature search performed by the NICE guidelines committee. Details of the literature search are reported below. The TSC drew up the tables of evidence, summarized the findings of the included articles and supported the invited experts in the analysis of the relevant selected literature. Tables of evidence comprised details on the source, eligibility, study design, characteristics of participants, interventions, outcomes, and results of included studies.

The CC organizers and the TSC selected the members of the Independent Panel (IP) following the same criteria used for the selection of EP. The IP included the following members: three pediatric anesthesiologists working in the PICU setting, an adult anesthesiologist, three pediatric emergency physicians, an adult emergency physician, a nurse working in the pediatric ED, a nurse working in the general ED, a clinical pharmacologist, a pharmacist, a pediatric orthopedic surgeon, a pediatric surgeon, a member of the Cochrane Collaboration, a bioethicist, a family pediatrician and a parent representative. The list of all the members of the TSC, the EP and the IP and their professional roles are available at request.

Involvement of citizens/patients/relatives or their representatives is recommended by the methodological standards followed for the development and conduct of the CC [34]. During the discussion the role of the bioethicist and the parents’ representative was to protect children’s interest, looking at PSA from a different perspective. Their role in the discussion was to draw the attention on specific topics, such as, effective communication on the procedure and PSA in the medical consent process. However, non-physicians had no voting rights and were not allowed to discuss medical details during the project. They had been instructed about this before the beginning of the project.

Of the authors of this manuscript L.DD, L.B., F.DI were part of the Independent Panel; E.B. was the Chair of the Independent Panel; I. Sh and B. K. were part of the EP.

The IP had the following tasks: to attend all the presentation and discussion sessions at the conference, to re-examine the evidence, to draft the final consensus document and present it to all the participants to the CC at the last conference session.

Official representatives of scientific societies and other professional experts in pediatric PSA were invited to participate to the CC in the audience. They did not cover any of the above-described roles in order to develop a document “super partes”.

The CC was held on January 16–17, 2017 in Florence, Italy. The drafting and refinement of the consensus recommendations continued in the following months. The final document was then presented to the relevant scientific societies for endorsement.

### Literature search

An electronic literature search was carried out for questions 1, 2, 3, 4, 5 in MEDLINE and EMBASE between 03/06/2012 (end-date of the last literature search for the NICE guidelines) and 04/08/2016.

The selection of relevant articles was conducted by two independent members of the TSC, following pre-defined inclusion criteria, described below. In case of disagreement, other two members independently revised the articles and a final agreement was reached. The reference lists of relevant studies were also reviewed to identify additional eligible studies.

The selection of relevant evidence and summary of selected articles in the evidence tables were performed between August and November 2016. The results of the selection and summary processes were then sent to the EP and IP, to prepare for the presentation and discussion sessions at the CC in January 2017.

The adopted research syntax on PubMed was: (sedat* [ALL FIELD] OR [(minimal OR light) AND (anesthesia OR anaesthesia)] [ALL FIELD] OR conscious sedation [MESH] OR deep sedation [MESH] OR dental anxiety [MESH]) AND (child* [ALL FIELD] OR child [MESH] OR infan*[ALL FIELD] OR infant [MESH] OR [baby OR babies] [ALL FIELD] OR adolescen* [ALL FIELD] OR adolescent [MESH] OR [pediatric* OR paediatric*] [ALL FIELD]). The same research syntax, with necessary changes, was used for EMBASE.

With respect to the question on dexmedetomidine, it was necessary to use a separate search strategy, without time limits, as this medication was not included in the NICE guidelines. The research syntax for dexmedetomidine on PubMed and EMBASE was the following: (sedat*[ALL FIELD] OR [(minimal OR light) AND (anesthesia OR anaesthesia)] [ALL FIELD] OR conscious sedation [MESH] OR deep sedation [MESH] OR dental anxiety [MESH]) AND (child* [ALL FIELD] OR child [MESH] OR infan*[ALL FIELD] OR infant [MESH] OR [baby OR babies] [ALL FIELD] OR adolescen* [ALL FIELD] OR adolescent [MESH] OR [pediatric* OR paediatric*] [ALL FIELD]) AND dexmedetomidine [ALL FIELD].

All searches were limited to studies including Human Subjects, written in English or Italian and including a study population age range from birth to 18 years.

For questions 6, 7 and 8, no systematic search was carried out; however, a separate review of the literature was conducted by individual experts. We adopted this strategy because of the specific nature of the questions for which we did not expect evidence to be available. Only for questions 6, 7 and 8, each expert developed their own search strategy with the purpose of being as sensitive as possible and including all kinds of useful documents.

Studies selected for inclusion were relevant systematic reviews, meta-analysis, randomized controlled trials and observational studies. Studies that included both children and adults were included if pediatric data could be analyzed separately. We selected studies conducted in the ED setting or in mixed settings including ED.

## Results

### The literature search

**Fig. 1:**
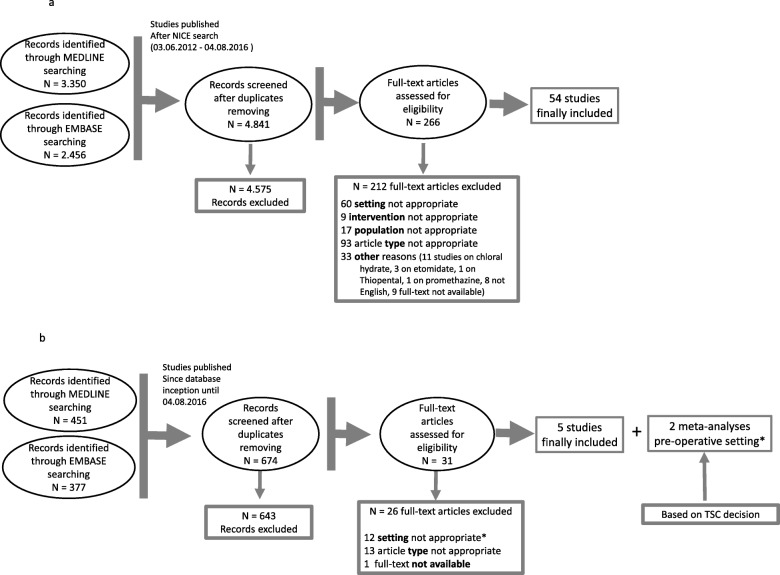
Flow chart of selection of relevant literature for **a** medications included in the NICE guidelines **b** for the additional medication dexmedetomidine

The literature search identified 3350 records in PubMed and 2456 in EMBASE. After duplicates were removed, 4841 records were screened. Overall, 266 studies were identified as potentially eligible for inclusion after screening of titles and abstracts. Finally, 54 studies were included based on full text reading (Fig. 1a).

With respect to the search on dexmedetomidine 451 studies were identified in PubMed and 377 in EMBASE. After duplicates were removed, 31 studies were screened. Following full text reading, 5 studies were finally included. Given the limited number of retrieved studies and the absence of a previous NICE literature analysis on dexmedetomidine, the TSC decided to also include the only published 2 meta-analyses at the time, although they analyzed the use of dexmedetomidine as premedication in the pre-operative setting (Fig. 1b).

Following the selection process a total of 61 studies were included and discussed at the CC. The number and design of studies included for each clinical question are reported in Table 2.

**Table 2:** Number of studies selected for each clinical question and study design (literature retrieved from 03/06/2012 to 04/08/2016)

Clinical questions	RCT ^**a**^	Observational studies ^**a**^	Systematic review ^**a**^
Q1 Pre-assessment and fasting	1	2	–
Q2 Pharmacological Treatment	15	30	5
- Midazolam	3	4	2
- Fentanyl	1	5	1
- Nitrous Oxyde	1	5	3
- Ketamine	12	12	2
- Propofol	–	7	2
- Dexmedetomidine	1	4	2
Q3 Monitoring	1	2	–
Q4 Check List	–	–	–
Q5 Training	1	4	–
Q6 Psychological strategies and non-pharmacologic techniques	No systematic search		
Q7 Emergency Medicine Physicians	No systematic search		
Q8 Impact on Organization and Hospital Admissions	No systematic search		

^a^ the studies included could report on pooled/summary data on more than one medication

### The conduct of the consensus conference

During the first day, the expert speakers and discussants presented and favor the debate on the evidence available on the clinical questions for each of the eight themes. At the end of the discussion, the IP met separately to reach a consensus on each topic through discussion. In this way, a method similar to a Quaker based-consensus method was used [35]. During the conference multiple concerns and information were shared until the sense of the group was clear, thanks to the expert speakers and the discussants assigned to each topic. Discussion involved active listening and sharing information. At the IP meeting the facilitator limited the number of times a member asked to speak to ensure that each member was fully heard. Differences of opinions were resolved by discussion and disagreements were identified to push discussion deeper. The facilitator summarized the key points of the discussion, asking if there were other concerns, and proposing a “minute” of the agreed upon recommendation. Recommendations were polished until unanimous agreement was reached. A first draft of recommendations for each clinical question was developed. During the second day the provisional recommendations were illustrated to all participants: the EP, the invited Audience, the CC organizers and the TSC. Feedback from participants was sought and incorporated as appropriate.

In the next weeks the IP worked on refining the list and content of the recommendations. In case consensus was not reached a blind electronic discussion was carried out and the recommendations polished until unanimous agreement was later achieved.

The final content of the consensus document was finalized in June 2017.

### The final consensus conference recommendation document

The final document was published in Italian in December 2017. It included a total of 86 recommendations: 14 on the pre-assessment, 31 on the efficacy and safety of sedation medications (midazolam, opioids, nitrous oxide at 50%, ketamine, propofol, and dexmedetomidine), 8 on monitoring, 8 on the checklists for equipment and discharge, 18 on training and development of a curriculum at the individual institution/hospital level, 6 on non-pharmacologic techniques, and one on PSA in general EDs. For the clinical question on the impact on hospitalizations and resource use no specific recommendation could be crafted due the lack of specific data in the literature or from national experience.

The full list of recommendations is reported in Additional file 1. A detailed explanation of the justification behind each recommendation is available in the extended Italian document, which is open access (see below).

### Knowledge dissemination

The Consensus document has been made available for free download at http://www.meyer.it/index.php/didattica-e-formazione/documenti in Italian language.

The Consensus recommendations have been presented at the national conferences of the Italian Emergency Medicine and Pediatric Emergency Medicine societies in 2018.

### Endorsement

The document has been endorsed by the Italian Society of Pediatric Emergency Medicine (SIMEUP), and the Italian Society of Emergency Medicine (SIMEU), according to their internal procedures, and advertised on their website.

## Discussion

We reported the process and results of the first consensus document in Italy on pediatric PSA in the ED setting to be used as a reference and a guide for the development and implementation of safe and effective PSA across Italian pediatric and general EDs, in agreement with individual institution policies and protocols.

The Consensus document has several strengths.

First, the whole process that led to the CC and the development of the document followed a rigorous methodology [34] that warranted transparency and was inclusive of our country diversities in terms of practice, geography and healthcare settings.

Second, the document was the result of the contributions of a thorough multidisciplinary and multi-professional group of experts, ensuring inclusiveness and broad professional and end-user representation, to facilitate a satisfactory level of agreement at a national level.

This document represents a first step in the attempt to establish and implement national standards for pediatric PSA in Italian ED, while recognizing the differences between institutions and the need to translate these recommendations into local protocol and policies at individual institutions. In the absence of a nationally recognized curriculum and training each institution is mandated with the task of certifying the skill-set and competencies of their emergency physicians and pediatric emergency physicians providers, as well as the maintenance of the above over time, in order to warrant safe and effective PSA to children in the ED. While Pediatric Elective Sedation Services run by pediatricians are well established entities in some tertiary-care pediatric centers in Italy [31, 36, 37], the practice of pediatric PSA in the ED is often hampered by the fear of potential sedation adverse events, neglecting that emergency physicians and PEM physicians have the skill-set required to handle airway, ventilation and cardiovascular emergencies, as these competencies are required to cover their professional role. However, in order to create a safe PSA environment in the ED the training of nursing staff is also paramount. The experience of colleagues working in the elective pediatric PSA setting will be valuable to share ideas and collaborate on training requirements and institution-based pathways. Similarly, the collaboration with the intensivists and anesthesiologists will be important not only for the training of providers, but also to establish a shared back-up plan to best handle rare severe adverse events.

Third, the document makes specific reference to general EDs, where most of children are actually seen in our country. The involvement of emergency physicians representatives in the consensus and the endorsement of the Italian Society of Emergency Medicine represent an important step forward to ensure that standards of care are provided to the great majority of Italian children across ED settings and specialists, thus reducing disparities in the access to high-quality care.

The CC and the development of the final document could count on and benefit from the contribution and support of internationally recognized experts in the field of pediatric PSA in the ED.

The result of our CC, however, has to be interpreted in light of some limitations. We have followed the Italian National Methodological standards for the development and conduct of the CC [34]. We did not use formal tools to correct for potential higher influence/weight of some members of the IP in the development of the final recommendations. We have used the unblinded methodology to achieve consensus on the recommendations. We understand that this could have led to a higher influence of more authoritative IP members on the content of recommendations [35]. Neverthless, we believe that the role of the facilitator and the presentation of the provisional recommendations to all conference participants during the second day may have mitigated this potential bias. In addition, according to the methodological standards followed for conduct the CC, we did not grade the recommendations according to their strength, which relates to the quality of the available evidence, based on the GRADE methodology [38]. We summarized the results of all relevant studies in evidence table format, which are available in Italian, on request. We assessed randomized controlled trials for their internal validity (based on the potential for performance, attrition and detection bias), clinical relevance and external validity. Observational studies were assessed based on the EQUATOR reporting guidelines [39]. These are reporting checklists rather than quality checklists and do not systematically assess possible biases [40].

### Future directions and challenges

We recognize that the development of the consensus document is only the first step towards the nation-wide implementation of safe and effective pediatric PSA practice in the ED. In addition, being the first initiative in the field, there is certainly room for improvement in the process, organization and conduct of future editions. In the meantime several other steps need to be made to develop a mature learning national PSA system. First of all, a national database should be established following the North-American models [15, 41] to monitor the quality of performed PSA and to document adverse events [3, 41–45]. It will also be important to establish a national pediatric PSA network [44, 46] to give the opportunity to PEM and EM providers to share their experience, knowledge and practice with respect not only to PSA per se, but also to its implementation at individual institutions, including challenges and successful strategies.

As for the consensus document, it will be periodically updated (the next update is scheduled for 2020) and refined aiming for a broader endorsement from scientific societies of other relevant specialties. While endorsed by the adult and pediatric emergency medicine societies, we hope a broader endorsement and agreement from multi-professional societies could be reached in the future. As demonstrated by those countries where PEM has long been formally recognized and has long had its individual identity, the process to achieving a shared multidisciplinary endorsement and support of PSA practice in the ED by non-anesthesiologists may be long and complex. Although we have important successful examples from these countries and increasing high quality evidence base to help expedite the process of implementing safe and effective PSA in the ED in countries where PEM is not formally recognized yet, historical, cultural and discipline-related impediments may still stand in the way of best patient care. However, in light of recent European initiatives, such as the PROSA conference held in Maastricht in November 2018 [47], aimed at sharing safe and effective PSA practice in several different settings with expert professionals from multiple disciplines and countries, we believe time has come to join forces to promote and facilitate a productive dialogue between supporters and opposers of pediatric PSA in the ED.

## Conclusion

The results of the first multidisciplinary consensus conference on ED-PSA in Italy in children provided up-to-date national guidance. Children have the right to receive the standard of care when undergoing painful and/or distressful procedures in the ED. The road to achieving the goal of effective implementation of pediatric PSA in the ED in countries where PEM is not formally yet recognized may still be hindered by several obstacles despite the successful examples of other countries and growing high-quality evidence. The Consensus Conference document represents a first step towards this goal in our country and may serve as an improvable frame for pediatric societies or other countries willing to or in the process to move in the same direction.

## Supplementary information

**Additional file 1.** List of recommendations.

## Data Availability

Not applicable.

## Abbreviations

CC: Consensus Conference
ED: Emergency Department
EM: Emergency Medicine
EP: Expert Panel
IP: Independent Panel
NICE: National Institute for Health and Care Excellence
PEM: Pediatric Emergency Medicine
PSA: Procedural sedation and analgesia
SIMEU: Italian Society of Emergency Medicine
SIMEUP: Italian Society of Pediatric Emergency Medicine
TSC: Technical Scientific Committee
