# Supplementary material for: The development of a Consensus Conference on Pediatric Procedural Sedation in the Emergency Department in Italy: from here where to?
Source: Ital J Pediatr. 2020 May 1;46:57. doi: 10.1186/s13052-020-0812-x (PMC7195721; doi:10.1186/s13052-020-0812-x)
Supplement: Supplementary file 1 — Additional file 1. List of recommendations. [file 13052_2020_812_MOESM1_ESM.docx]

**Additional file 1**

**LIST OF RECOMMENDATIONS**

All the following recommendations apply to children and young people under the age of 18 undergoing diagnostic and therapeutic procedures under procedural sedation and analgesia (PSA) techniques  in the Emergency Department (ED).

**QUESTION 1**

Defining patients eligible for PSA in the ED by non-anesthetists and the role of fasting.

- 1. *Which factors should be assessed to justify the use of PSA, rather than no sedation or general anesthesia?*

**Recommendation 1**

An evaluation of the following is recommended in order to establish the feasibility of PSA in the ED: type of procedure to be carried out, the patient’s current medical conditions, weight, past/underlying diseases -including any side effects associated with previous sedation or anesthesia- a physical examination, aimed especially at assessing the airways and cardiac and respiratory conditions, psychological conditions and development.

**Recommendation 2**

It is recommended that the choice of PSA technique to use be based on the type of procedure, the degree of sedation desired, the presence or otherwise of possible contraindications and the potential side effects.

**Recommendation 3**

It is recommended to consult a specialist for PSA in patients with potential problems with airway patency or breathing, for children with co-morbidities, those with ASA ≥ 3, as well as with newborn and young infants.

**Recommendation** **4**

It is recommended that the following always be available during PSA:

- at least one healthcare practitioner qualified in the management and monitoring of sedation in children and adolescents
- the necessary equipment for resuscitation and monitoring (with immediate access).

**Recommendation 5**

It is advised to adapt the consent document based on the specific protocols and procedures of individual institutions, which should be agreed upon by a local multidisciplinary team of experts.

- 1. *What validated tools should be used to support assessment?*

**Recommendation 6**

It is recommended to carry out a standardized pre-PSA evaluation that includes the following elements from medical-history for the purpose of identifying potential risk factors:

- allergies
- medicines administered
- past reactions to anesthesia and/or sedation
- pre-existing conditions
- snoring
- sleep apnea
- recent respiratory infections
- ASA class
- time of the last intake of solids and liquids
- physical examination including the Mallampati classification
- vital parameters (HR, SpO_2_, RR and BP)

*1.3 Who should make the assessment and how should the assessment be recorded?*

**Recommendation** **7**

It is recommended that the pre-PSA evaluation be carried out by the adequately trained ED physician who will perform the PSA.

**Recommendation** **8**

It is recommended that the pre-PSA evaluation be recorded in the medical record.

- 1. *How should the consent for PSA be obtained?*

**Recommendation** **9**

It is recommended to obtain and document consent for PSA by providing verbal or written information. The consent must be obtained by the doctor who performs the PSA.

- 1. *Should fasting versus no fasting be implemented to prevent adverse outcomes?*

**Recommendation** **10**

Fasting is not recommended in the case of procedures that require minimal sedation or sedation with 50% nitrous oxide premixed with O_2_ during which the patient maintains verbal contact with the provider.

**Recommendation** **11**

In the case of elective procedures in moderate and deep PSA, it is recommended for fasting to be carried out in accordance with the 2-4-6 rule.

**Recommendation** **12**

In the event of emergency procedures in a child that has not fasted, it is recommended that the decision to carry out the procedure in PSA be made after an analysis of the risks/benefit ratio, assessing the need for fasting on a case-by-case basis, with a less restrictive attitude than the 2-4-6 rule.

**Recommendation** **13**

If after the analysis of the risks/benefit ratio it has been decided to sedate a non-fasting patient in the ED, for a non-elective procedure it is advised to:

- prefer minimal or moderate sedation
- use additional techniques to obtain analgesia and patient cooperation (distraction, guided imagery, video games, topical and locoregional anesthesia)
- use drugs with less risk of depression of the protective reflexes of the airways such as ketamine

**Recommendation** **14**

It is recommended for the evaluation of fasting to be an integral part of the pre-PSA evaluation, without considering the lack of fasting as an absolute contraindication to performing PSA.

**QUESTION 2**

Evaluating the drugs, which can be used by non-anesthetists for PSA in the ED, as well as the dosages, possible combinations and various administration routes.

**Recommendation** **15**

It is recommended to prefer drugs with a rapid half-life, with the availability of reversal agents: caution is advised when considering the use of drugs in combination, given the increased risk of deep sedation and potential side effects.

**Recommendation** **16**

In the event of a combination of drugs for PSA it is advised to take the risk of synergistic effects into account and to appropriately reduce the dosage of the individual drugs.

**Recommendation** **17**

In the approach to a child who has to undergo a painful procedure it is always advisable to assess the use of topical anesthesia.

*2.1 Is* ***midazolam*** *(with or without: analgesia, another drug or psychological techniques) effective and safe for sedation (at minimal, moderate, and deep levels) in comparison with usual care, with analgesia alone, with another sedation drug, with psychological techniques or with general anesthesia?*

**Recommendation** **18**

It is recommended to use midazolam as a first-line drug for PSA.

**Recommendation** **19**

It is recommended to use midazolam as a first-line drug for short-duration diagnostic imaging (e.g. CT scan) in children requiring sedation.

**Recommendation** **20**

It is recommended to use oral or intranasal midazolam in combination with local anesthetics if it is necessary to control anxiety as well as pain.

**Recommendation** **21**

It is recommended to use oral or intranasal midazolam, if necessary also in combination with fentanyl, for moderately painful procedures (for example, suturing of wounds or orthopedic manipulations).

**Recommendation** **22**

It is recommended to use the mucosal atomizing device (MAD) for intranasal administration of midazolam and to add 0.1-0.2 ml (variable dead space depending on the MAD used) to the total dose to be administered.

**Recommendation** **23**

It is recommended to use intravenous midazolam, if necessary in combination with fentanyl, for moderate or severe painful procedures (for example, orthopedic manipulations) in which 50% N_2_O associated with oral or intranasal midazolam, or 50% N_2_O associated with fentanyl or ketamine, are not indicated

**Recommendation** **24**

It is recommended to use midazolam for PSA at the dosages shown in the attached table.

| Route of Administration | Dose | Max | Onset | Duration |
| --- | --- | --- | --- | --- |
| IV | 0.1-0.2 mg/kg | 5 mg | 1 min | 30-45 min |
| IN | 0.3-0.7 mg/kg | 15 mg | 10-15 min | 30-45 min |
| PO | 0.5-0.8 mg/kg | 15 mg | 15-30 min | Up to 60 min |

*2.2 Are* ***opioids*** *(with or without: analgesia, another drug or psychological techniques) effective and safe for sedation (at minimal, moderate, and deep levels) in comparison with usual care, with analgesia alone, with another sedation drug, with psychological techniques or with general anesthesia?*

**Recommendation** **25**

It is recommended to use fentanyl as the opioid of choice for PSA.

**Recommendation** **26**

It is recommended to use intranasal or intravenous fentanyl in combination with a sedative drug for moderately painful procedures (for example, reduction of fractures/dislocations, drainage of abscesses, removal of foreign bodies, suturing of complex wounds).

**Recommendation** **27**

Even if there are no reported cases of chest wall rigidity in the procedural sedation literature and the event only occurred in the anesthesiology setting at higher medication doses, we suggest a slow IV administration of fentanyl.

**Recommendation** **28**

Caution is advised when considering the combination of fentanyl with other drugs, given the greater risk of deep sedation and side effects.

**Recommendation** **29**

It is advisable to use the MAD for the intranasal administration of fentanyl and to add 0.1-0.2 ml (variable dead space on the basis of the MAD used) to the total dose to be administered.

**Recommendation** **30**

It is recommended to use fentanyl for PSA at the dosages shown in the table attached.

| **Route of administration** | **Dose** | **Onset** | **Duration** |
| --- | --- | --- | --- |
| IV | 1-2 mcg/kg | 1 min | 20-30 min |
| IN | 1-2 mcg/kg | 2-5 min | 20-30 min |

*2.3 Is* ***50% nitrous oxide premixed with 50% O2*** *(with or without: analgesia, another drug or psychological techniques) effective and safe for sedation (at minimal, moderate, and deep levels) in comparison with usual care, with analgesia alone, with another sedation drug, with psychological techniques or with general anesthesia?*

**Recommendation** **31**

It is recommended to use 50% nitrous oxide premixed with 50% O_2_ as first-line treatment in cooperating children and adolescents requiring procedures with minimal-mild pain.

**Recommendation** **32**

It is recommended to use 50% nitrous oxide premixed with 50% O_2_, combined with fentanyl, for moderately painful procedures (for example, suturing of wounds or orthopedic manipulations) for which midazolam (either oral or intranasal) is not indicated.

**Recommendation** **33**

It is advised to consider “continuous flow” administration of 50% nitrous oxide premixed with 50% O_2_ in children under the age of 5-6, and “on demand” in older children.

*2.4 Is* ***ketamine*** *(with or without: analgesia, another drug or psychological techniques) effective and safe for sedation (at minimal, moderate, and deep levels) in comparison with usual care, with analgesia alone, with another sedation drug, with psychological techniques or with general anesthesia?*

**Recommendation** **34**

It is recommended to administer ketamine intravenously or intramuscularly for moderate to severe painful procedures (for example, orthopedic manipulations), in which the use of 50% nitrous oxide premixed with 50% O_2_ and IV fentanyl or IV midazolam and IV fentanyl are inadequate.

**Recommendation** **35**

The routine use of ketamine is not recommended for painless diagnostic imaging procedures.

**Recommendation** **36**

It is not recommended to combine ketamine with midazolam to prevent the onset of emergence reaction.

**Recommendation** **37**

It is not recommended to combine ketamine with atropine to prevent the adverse events related to drooling.

**Recommendation** **38**

It is recommended to use ketamine for PSA at the dosages shown in the table attached.

| **Route of Administration** | **Dose** | **Onset** | **Duration** |
| --- | --- | --- | --- |
| IM | 4-5 mg/kg | 5 min | 15-45 min |
| IV | 1-2 mg/Kg | 1 min | 15-20 min |

*2.5 Is* ***propofol*** *(with or without: analgesia, another drug or psychological techniques) effective and safe for sedation (at minimal, moderate, and deep levels) in comparison with usual care, with analgesia alone, with another sedation drug, with psychological techniques or with general anesthesia?*

**Recommendation** **39**

It is recommended to use propofol as a third-line drug for sedation during painful procedures.

**Recommendation** **40**

It is recommended for propofol to be used strictly by professionals with appropriate skills in the management of deep sedation, after the definition of an internal policy together with the anaesthesiologists.

**Recommendation** **41**

It is recommended to use propofol as a single agent in the ED for diagnostic imaging (CT/MRI) or for minimally painful procedures in which it is combined with topical or local anesthesia that cannot be performed with the use of midazolam alone, bearing in mind that propofol has a low safety margin.

**Recommendation** **42**

It is recommended to use propofol in combination with an adequate analgesic (fentanyl, ketamine or locoregional anesthesia) for PSA in moderate-severe painful procedures if the use of ketamine (intravenous or intramuscular) or midazolam and fentanyl are inadequate.

**Recommendation** **43**

It is recommended to use propofol for PSA at the dosages shown in the table attached.

| **Route of Administration** | **Dose** | **Onset** | **Duration** |
| --- | --- | --- | --- |
| IV | 1-2 mg/Kg | 30-90 sec | 5-15 min |

*2.6 Is* ***dexmedetomidine*** *(with or without: analgesia, another drug or psychological techniques) effective and safe for sedation (at minimal, moderate, and deep levels) in comparison with usual care, with analgesia alone, with another sedation drug, with psychological techniques or with general anesthesia?*

**Recommendation** **44**

It is recommended to use dexmedetomidine as single drug in the ED for painless diagnostic imaging, in which immobility is necessary, and for performing electroencephalograms.

**Recommendation** **45**

It is recommended to use dexmedetomidine for PSA at the dosages shown in the table attached.

| **Route of Administration** | **Dose** | **Onset** | **Duration** |
| --- | --- | --- | --- |
| IM | 1-4 mcg/kg | 10-15 min | 45 min |
| IV | 1-3 mcg/kg | 10-15 min | 30-45 min |
| IN | 2-4 mcg/kg | 45 min | 60-90 min |
| buccal | 3-4 mcg/kg | 45-60min | 60-90 min |

**QUESTION 3**

Defining the systems and timing of the monitoring and assessment tools for PSA, to be used in the ED depending on the type of PSA carried out.

**Recommendation** **46**

It is not recommended to use any monitoring, apart from clinical observation, in the event of minimal sedation, provided verbal contact is maintained with the child, otherwise oximetry can be used.

**Recommendation** **47**

Monitoring with oximetry and capnograph is recommended with moderate and deep sedation.

**Recommendation** **48**

It is recommended to use monitoring with oximetry, capnograph and cardiomonitor in patients sedated with propofol and to monitor their arterial blood pressure every 5 minutes or more frequently if low blood pressure is detected.

**Recommendation** **49**

It is recommended for monitoring to be carried out by appropriately trained health professionals and, in the case of deep sedation, by a specialist, i.e. in a 1:1 ratio.

**Recommendation** **50**

It is only recommended to consider discontinuing the monitoring when the child presents:

- patent airways
- protective reflexes
- stable hemodynamics
- resumption of the state of pre-sedation consciousness

**Recommendation** **51**

It is advised to use the modified Ramsey scale* for assessing the depth of sedation during the procedure.

**In the next update of the consensus document it will be proposed to replace the Ramsey Scale with the Pediatric Sedation State Scale (PSSS), which was published after the literature search end date. PSSS has several strengths over the Ramsey Scale and is more appropriate to assess the effectiveness, quality and safety of PSA provided in the ED.*

**Recommendation** **52**

It is advised to evaluate amnesia and the degree of patient and caregiver satisfaction before discharge.

**Recommendation** **53**

It is recommended to use instruments that are specific for the evaluation of pain and anxiety for children with special needs who require PSA in the ED (revised FLACC).

**QUESTION 4**

Defining the checklist for the management of PSA in the ED: checklist for material, checklist for discharge.

**Recommendation 54**

It is recommended for the following equipment to be present in the ED, in the room where the procedures are carried out or in adjacent rooms, and it must be readily available:

- Face masks (various sizes)
- Breathing bag and valve set
- Oropharyngeal airways (various sizes)
- Nasopharyngeal airways (various sizes)
- Laryngeal mask airways (1, 1.5, 2, 2.5, 3, 4, and 5)
- Laryngoscope handles (with extra batteries) and laryngoscope blades (with extra light bulbs) straight (Miller) numbers 1, 2, 3 and curved (Macintosh) numbers 1, 2, 3 and 4
- Endotracheal tubes 2.5, 3.0, and 3.5 mm internal diameter uncuffed and 3.0, 3.5, 4.0 4.5, 5.0, 5.5, 6.0, 7.0, and 8.0 mm internal diameter cuffed
- Stylettes (appropriate sizes for endotracheal tubes)
- Surgical lubricant
- Suction catheters (appropriate sizes for endotracheal tubes) and Yankauer-type suction
- Nasogastric tubes
- Nebulizer with medication kits
- Gloves (sterile and non-sterile, latex free)
- Drugs: Albuterol for inhalation, amiodarone, atropine, Dextrose, Chlorphenamine, Diazepam, Epinephrine (1:1000, 1:10 000), Flumazenil, Lidocaine, Methylprednisolone, Naloxone, Oxygen, Fosphenytoin, Rocuronium, Sodium bicarbonate, Succinylcholine
- Pulse oximeter
- Cardiac monitor
- End-tidal carbon dioxide monitor with oral/nasal cannula
- Defibrillator with pediatric pads

**Recommendation 55**

It is recommended that the equipment be checked before each PSA.

**Recommendation 56**

It is advised to refer to the acronym SOAP-ME as a reminder to check the equipment.

**Recommendation 57**

It is recommended to use the Modified Aldrete scale for discharging the patient undergoing PSA in the ED.

**Recommendation 58**

It is recommended to adequately manage any nausea, vomiting or pain before discharge.

**Recommendation 59**

It is recommended to consider the risk of dehydration and hypoglycemia in the child with prolonged fasting, especially if under the age of one year.

**Recommendation 60**

In the case of children who have undergone sedation with drugs with a long half-life being transported in a car seat with only one person accompanying them, it is recommended to postpone discharge until they have completely recovered.

**Recommendation 61**

It is advised that at the time of discharge the family be provided with written instructions for management during transport and at home.

**QUESTION 5**

Defining the training path necessary for a nurse, pediatrician and ED physician for safely performing PSA in the ED and institutionalize such training at a national level with the creation of a specific curriculum.

*5.1 What generic and specific skills are required for different team members and for different levels of sedation? What training and competences are required?*

**Recommendation 62**

It is recommended for the medical personnel to have successfully completed the advanced resuscitation courses such as PALS/EPALS, and for the nursing personnel to have successfully completed the intermediate or advanced resuscitation courses like EPILS/PALS/EPALS.

**Recommendation 63**

It is recommended for all staff, doctors and nurses, assigned to the administration of pediatric PSA in the ED, to receive specific training. In particular, they must have theoretical and practical training in the:

- evaluation of pediatric patients
- pharmacology and physiology of sedative drugs
- correct performance of the sedation chosen
- knowledge of the monitoring systems
- assessment of resumption of normal functions after sedation
- recognition and management of any complications
- management of the airways
- performance of cardiopulmonary resuscitation

**Recommendation 64**

A differentiated training course is recommended for doctors who perform minimal-mild PSA in the ED (midazolam oral and intranasal, intranasal fentanyl, 50% nitrous oxide premixed with 50% O_2_, intranasal fentanyl in combination with 50% nitrous oxide premixed with 50% O_2_) and moderate-deep PSA (ketamine, propofol and combination drugs).

**Recommendation 65**

It is recommended for all the points of recommendations 62 and 63 to be complied with for minimal-mild PSA.

**Recommendation 66**

During moderate-deep sedation in the ED it is recommended for a doctor to be assigned exclusively to the sedation in order to be able to observe the patient, monitor the vital signs, the airway patency, the adequacy of the ventilation, and to administer the sedation medications or supervise their administration.

**Recommendation 67**

It is recommended for the doctor performing moderate-deep sedation to be skilled in resuscitation, in particular to know how to manage apnea, laryngospasm and airway obstruction. The skills required include airway opening maneuvers, bag-mask ventilation, the use of supraglottic devices, and the ability to perform orotracheal intubation and cardiovascular resuscitation.

**Recommendation 68**

It is recommended for the physician performing moderate-deep sedation to have attended certified basic life support courses, to have the skills for performing minimal-mild sedation, to have attended specific theoretical and practical courses on moderate-deep sedation, to have undergone supervised practice training, on the possible side effects and their management.

**Recommendation 69**

It is advised for the duration of training to depend on the ability of the trainee to acquire the necessary skills, however with a minimum number of procedures to be carried out under supervised practice, divided into 10 supervised procedures for the administration of ketamine and 20 supervised procedures for the administration of propofol.

**Recommendation 70**

It is advised for the training to be carried out, always under the supervision of an anesthesiologist or an expert ED physician in PSA, in the operating theatre and/or during elective procedures and/or during pain therapy and/or in the ED, even if the final decision for the site of the training will depend on the individual institution.

**Recommendation 71**

It is recommended for a training course to be held specifically for the nursing staff who perform PSA in the ED or assist the medical staff in the same: in particular, the nursing personnel must be competent in the techniques they carry out or which they assist, knowing how to constantly monitor the patient’s physiological parameters and assisting the physician in the management of the airway, while also possessing specific expertise for assisting in cardiovascular resuscitation.

**Recommendation 72**

It is recommended for the nurses who have been adequately trained in PSA to be an integral part of patient care during PSA in all its phases.

**Recommendation 73**

It is recommended for at least one of the figures performing PSA to be skilled in the placement of venous access.

**Recommendation 74**

It is recommended for the medical and nursing personnel to participate in periodic meetings on PSA in the ED, to be acquainted with the equipment, contribute to the drafting of protocols, and know how to coordinate their colleagues in case of emergency.

**Recommendation 75**

The filing of certifications acquired through retraining is recommended.

**Recommendation 76**

The consolidation of PSA skills is recommended through simulations, in particular with respect to the management of rare side effects.

**Recommendation 77**

It is advised for adequately trained and certified nursing staff to be able to autonomously administer 50% nitrous oxide premixed with 50% O_2_ as a single drug, in line with the medical prescriptions and subject to internal agreements of the individual hospitals.

*5.2 Who should train the nurses, doctors and pediatricians of the ED?*

**Recommendation 78**

It is recommended for the internal PSA training to be shared between the ED and the anesthesia department/ICU of the hospital.

**Recommendation 79**

It is advised for the theoretical and practical training courses, as well as the simulations and training to be carried out by medical personnel from different fields (anesthetists, ED physicians, ED/Intensive Care pediatricians) and nursing personnel with recognized and certified expertise.

**QUESTION 6**

Identify the strategies for effective implementation of the non-pharmacologic techniques for performing PSA in the ED.

*6.1 What standard psychological preparation, coping skills and strategies should be used?*

**Recommendation 80**

The personnel carrying out PSA should be aware of the anxiety conditions of the child and the family.

**Recommendation 81**

It is recommended to provide the information on the sedation/procedure to be performed to the child and family using a developmentally appropriate and child-friendly language, and to ensure a child-friendly environment, including the necessary equipment for non-pharmacologic techniques.

**Recommendation 82**

Family members should be welcomed, taking their psychological situation and involvement into account and wherever possible, considering them a potential resource for the success of the PSA itself.

*6.2 Can a combination of psychological techniques and sedative drugs help reduce the doses of sedatives?*

**Recommendation 83**

It is recommended that the approach to the child who has to undergo a painful or anxiety-inducing procedure always include non-pharmacologic therapy at every possible phase, from the preparation, to the performance of the procedure and the re-elaboration phase, considering it an integral part of the PSA itself.

*6.3 What instruments can be used to implement the use of the non-pharmacologic techniques?*

**Recommendation 84**

It is recommended for non-pharmacologic techniques to be taught in all the ED and that the personnel performing pediatric PSA in the ED to be adequately trained.

**Recommendation 85**

It is advised to use different instruments depending on the different ages of the children and to have tools such as “distraction kits” available (e.g. boxes containing interactive games, markers, soap bubbles…) or electronic devices and, wherever possible, to create a child-friendly environment.

**QUESTION 7**

Evaluating the provision of PSA in pediatric patients by emergency physicians in general EDs.

*7.1 In which way should PSA provided by adult ED physicians, from pre-assessment*

*to discharge, be distinguished from PSA administered by pediatricians/pediatric*

*emergency physicians and how should the differences be managed?*

**Recommendation 86**

All the recommendations applicable to PSA in the pediatric ED are also confirmed for the management of the pediatric patient in the General ED.

**QUESTION 8**

What impact could the performance of diagnostic and therapeutic procedures under PSA in the ED have on costs (for the patient, for the Institution, for the National Health System)?

**Recommendation 87**

There are no specific recommendations.
